# pH- and Near-Infrared Light Dual-Responsive Hydrogel Microneedles Incorporating Polydopamine-Based Curcumin-Loaded Nanoparticles for Antibacterial and Oxidative Injury Protection

**DOI:** 10.3390/polym18182269

**Published:** 2026-09-17

**Authors:** Xinyue Wang, Ranran Wang, Junyu Yi, Yan Wang, Gang Wei

**Affiliations:** 1 College of Chemistry and Chemical Engineering, Qingdao University, Qingdao 266071, China; xinyuewang0216@outlook.com (X.W.); wangranran0110@outlook.com (R.W.); 2 School of Polymer Science and Engineering, Qingdao University of Science and Technology, Qingdao 266042, China; junyuyi66@outlook.com; 3 Institute of Biomedical Engineering, College of Life Science, Qingdao University, Qingdao 266071, China

**Keywords:** microneedles, polydopamine, drug delivery, antibacterial activity, wound healing

## Abstract

Bacterial infection and persistent oxidative stress can impair wound healing and limit the efficacy of conventional antibacterial therapies. Here, we developed a pH- and near-infrared (NIR) light-responsive polymer hydrogel microneedle (PHMN) system by incorporating curcumin-loaded polydopamine particles (PDA@CUR) into photo-crosslinked methacrylated hyaluronic acid (HAMA) microneedles with a poly(vinyl alcohol) (PVA) backing layer. The synthesized CUR-loaded PDA nanoparticles (PDA@CUR) exhibited uniform spherical morphology, efficient CUR loading, and pH- and NIR light-responsive drug release. The fabricated PHMN/PDA@CUR patch displayed a well-defined array, sufficient mechanical strength, and efficient photothermal conversion under NIR irradiation. The patch could effectively inhibit both *E. coli* and *S. aureus*, with NIR irradiation further enhancing antibacterial activity. This PHMN-based system also exhibited favorable cytocompatibility and significantly alleviated H_2_O_2_-induced cellular oxidative injury. Overall, the PHMN/PDA@CUR patch integrate localized drug delivery, NIR-enhanced photothermal antibacterial activity, and cellular protection, providing an in vitro proof-of-concept platform for localized management relevant to infected wounds.

## 1. Introduction

Skin wounds complicated by bacterial infection remain challenges to treat due to persistent microbial colonization and biofilm formation, aggravate oxidative stress, and interfere with the normal progression of tissue repair [1,2]. In particular, the extracellular matrix of bacterial biofilms could restrict the penetration of antimicrobial agents and increase bacterial tolerance, whereas excessive reactive oxygen species (ROS) generated within the inflammatory microenvironment can impair cellular viability and further delay wound healing [3,4]. Therefore, more and more attention has been attracted on multifunctional wound-management systems capable of simultaneously providing effective local antibacterial activity and mitigating the detrimental effects of excessive oxidative stress [5].

Microneedles (MNs) have emerged as attractive platforms for localized wound treatment because their microscale needle arrays can overcome superficial biological barriers and facilitate the delivery of therapeutic agents directly to the wound interface [6,7]. Compared with conventional topical formulations, MN-based systems can improve local drug deposition while reducing dependence on passive diffusion across biological barriers [8]. Polymer hydrogel microneedles (PHMNs) are particularly attractive because their composition and mechanical properties can be readily tailored through polymer selection and crosslinking strategies [9,10]. For instance, hyaluronic acid (HA) and its chemically modified derivatives have been extensively explored for constructing PHMNs because of their excellent biocompatibility and suitability for biomedical applications [11], while poly(vinyl alcohol) (PVA) provides favorable film-forming and structural properties for polymeric delivery systems [12]. Moreover, incorporating polydopamine (PDA)-based functional components into PHMNs has demonstrated the feasibility of combining localized drug delivery with the intrinsic biological and photothermal properties of PDA matrix [9,13].

Curcumin (CUR), a naturally derived polyphenolic compound, exhibits antibacterial, antioxidant, and anti-inflammatory activities that are beneficial for wound healing [14,15]. However, its poor aqueous solubility and limited physicochemical stability restrict its direct incorporation into aqueous wound-treatment formulations [15]. Meanwhile, PDA-based nanotechnology provides an effective strategy to overcome these limitations [16,17]. Owing to its abundant aromatic and functional groups, PDA can load hydrophobic molecules such as CUR through noncovalent interactions, while its broad optical absorption enables efficient conversion of NIR irradiation into localized heat [18,19]. Previous studies have demonstrated that PDA-based CUR carriers improved the stability and delivery behavior of CUR and enabled pH-responsive release [18,20]. Furthermore, the PDA–CUR nanocomposites exhibited light-enhanced antibacterial activity [20], and the incorporation of PDA–CUR into hydrogels has enabled NIR-triggered CUR release together with photothermal antibacterial effects. These findings suggest that PDA can simultaneously function as a CUR carrier and a photothermal transducer, thereby providing a simple basis for integrating drug delivery and externally controlled antibacterial treatment [21].

Recent studies have further demonstrated the potential of PDA-containing photothermal PHMNs for the treatment of infected wounds [22,23]. Notably, a recently reported HA-based bilayer PHMN system integrated a PDA–indocyanine green nanoplatform with CUR to achieve NIR-activated antibacterial therapy [24]. Although this design highlights the therapeutic potential of combining photothermal and pharmacological antibacterial modalities, the incorporation of an additional photothermal agent increases the compositional complexity of the system and introduces additional interfaces that may affect drug loading, release, and material stability. In contrast, PDA possesses both strong NIR photothermal heating capability and abundant functional groups capable of interacting with CUR, suggesting that PDA@CUR nanoparticles could serve as an integrated dual-functional component without requiring a separate photothermal sensitizer.

**Figure 1:**
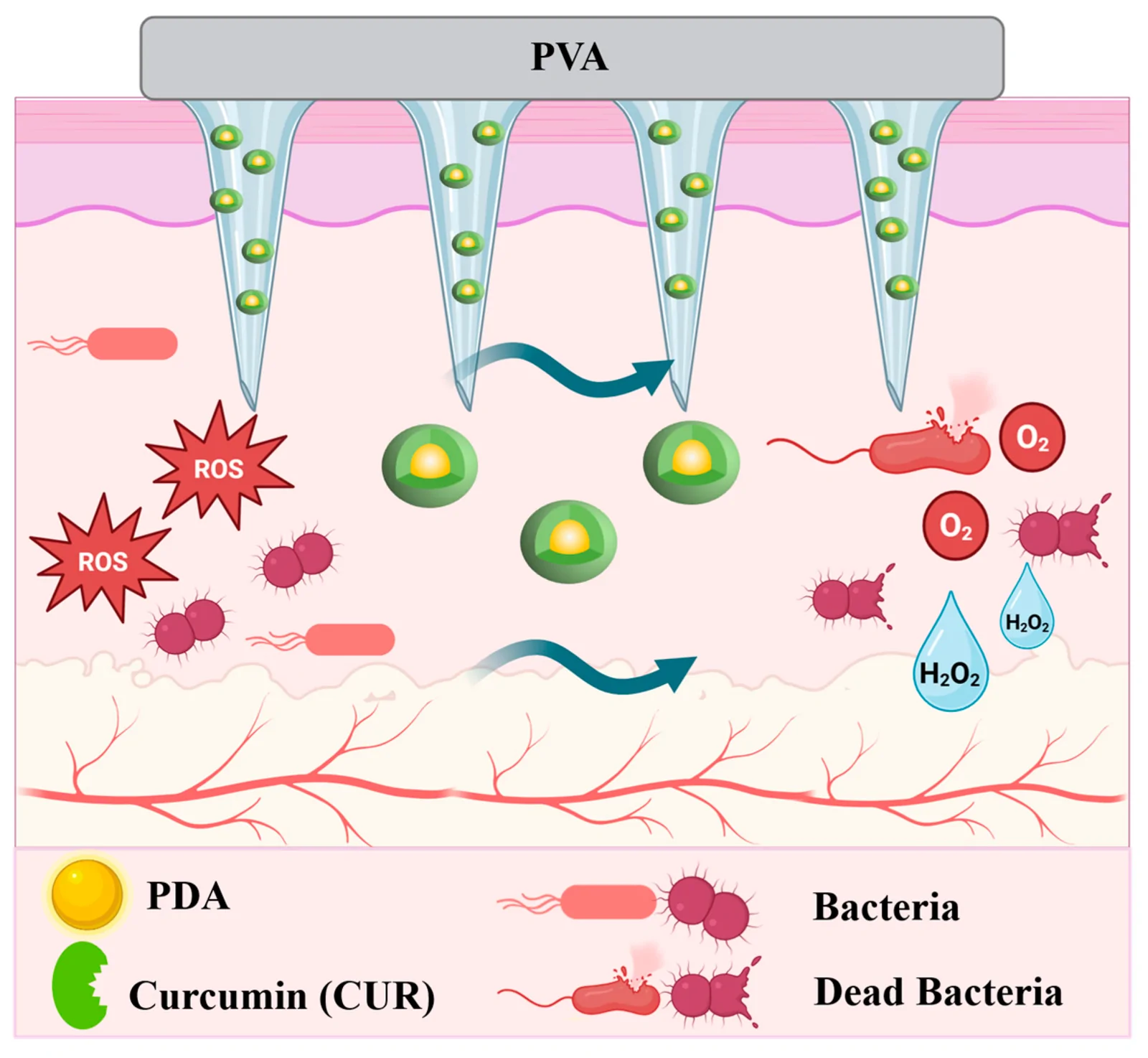
Construction of PHMN/PDA@CUR microneedle patch for antibacterial and cytoprotective applications. Image was created by Microsoft Office PowerPoint 2019.

In this study, CUR-loaded PDA were incorporated into photo-crosslinked methacrylated hyaluronic acid (HAMA) MN tips, with PVA employed as the backing layer, to form a multifunctional PHMN/PDA@CUR patch, as shown in Figure 1. The physicochemical characteristics and drug-loading performance of PDA@CUR, the morphology and mechanical properties of the resulting PHMN/PDA@CUR, and the pH- and NIR-responsive release behavior of CUR were systematically investigated. Its photothermal performance and antibacterial activity against *E. coli* and *S. aureus* were further evaluated, together with cytocompatibility and cellular protection under H_2_O_2_-induced oxidative injury. This PDA@CUR-integrated PHMN platform was designed to combine localized and stimuli-responsive CUR delivery with NIR-enhanced photothermal antibacterial activity, providing an in vitro proof-of-concept strategy for the localized management of infected wounds.

## 2. Materials and Methods

### 2.1. Materials and Reagents

Dopamine hydrochloride, CUR, PVA, lithium phenyl-2,4,6-trimethylbenzoylphosphinate (LAP), HA, methacrylic anhydride, H_2_O_2_, and NaOH were provided by the Shanghai Macklin Biochemical Co., Ltd. (Shanghai, China). HAMA was synthesized in the lab from HA and methacrylic anhydride according to the procedure described below. Ammonium hydroxide solution, absolute ethanol, methanol, dimethyl sulfoxide (DMSO), and phosphate-buffered saline (PBS) were bought from the Sinopharm Chemical Reagent Co., Ltd. (Shanghai, China). Ultrapure water was used throughout the experiments.

### 2.2. Preparation of PDA and PDA@CUR Nanoparticles

PDA nanoparticles were synthesized via a solution-based self-assembly method [25]. Briefly, 1 mL of aqueous ammonia solution (5 M) was mixed with ethanol (20 mL, 99%) and ultrapure water (35 mL), and mixed solution was maintained at 39 °C. After stirring for 30 min, dopamine (100 mg) was dissolved in the above solution, which was then gently stirred magnetically for 24 h to allow the oxidative polymerization of dopamine. It can be found that the color of the mixed solution altered from light brown to dark brown, indicating the polymerization and formation of PDA. The resulting PDA was collected by centrifugation, thoroughly washed several times with ultrapure water to remove unreacted species and impurities, and subsequently dried to obtain the PDA nanoparticles.

CUR-loaded PDA nanoparticles (PDA@CUR) were prepared through a solution-based drug-loading process. Briefly, 100 mg of dried PDA nanoparticles were redispersed in ultrapure water under magnetic stirring. CUR (75 mg) was dissolved in absolute ethanol to prepare a CUR stock solution, corresponding to a PDA/CUR mass ratio of 1:0.75 (*w*/*w*). The CUR solution was slowly added dropwise to the PDA dispersion under continuous stirring, while the final ethanol content was maintained below 10% (*v*/*v*) [26]. The mixture was gently stirred in the dark at 30 °C for 24 h to facilitate the incorporation of CUR into PDA through noncovalent interactions.

### 2.3. Fabrication of PHMN/PDA@CUR Patch

Methacrylated hyaluronic acid (HAMA) was synthesized from hyaluronic acid (HA) and methacrylic anhydride according to previously reported methods with minor modifications [27]. Briefly, HA was dissolved in ultrapure water to prepare a 1% (*w*/*v*) HA solution and stirred until completely dissolved. The solution was cooled in an ice bath, and methacrylic anhydride was slowly added dropwise under continuous stirring. During the reaction, the pH was maintained at 8.0–9.0 by the addition of NaOH solution to facilitate the methacrylation reaction. The reaction was continued under low-temperature conditions with continuous stirring, after which the resulting solution was transferred into a dialysis membrane and dialyzed against ultrapure water for 72 h, with the external water replaced periodically to remove unreacted methacrylic anhydride, methacrylic acid, and other low-molecular-weight impurities. The purified HAMA solution was subsequently freeze-dried and stored at 4 °C until further use.

For fabrication of the PHMN/PDA@CUR patch, the HAMA microneedle precursor containing PDA@CUR and LAP was introduced into a polydimethylsiloxane (PDMS) microneedle mold and subjected to vacuum treatment to facilitate complete filling of the needle cavities [28]. The filled mold was then exposed to UV irradiation (365 nm) for 5 min to induce LAP-mediated photocrosslinking of the HAMA network and form the microneedle tips. After formation of the HAMA-based needle layer, a PVA solution (10 wt%, 200 μL) was added onto the mold as the backing layer and dried at 37 °C until complete solidification [29]. The resulting PHMN/PDA@CUR patches were carefully demolded and stored under dry conditions until further characterization and biological evaluation.

### 2.4. Encapsulation Efficiency and Drug Loading

A calibration curve for CUR was established using a UV–visible spectrophotometer. Briefly, CUR was dissolved in absolute ethanol to prepare a stock solution and subsequently diluted to prepare standard solutions at concentrations of 5, 10, 20, 30, 40, and 50 μg/mL. The absorbance of each solution was measured at 425 nm, and the calibration curve was obtained by plotting absorbance against CUR concentration. The CUR concentration of unknown samples was calculated according to the corresponding linear regression equation.

After the loading reaction, the PDA@CUR dispersion was centrifuged to separate the particles from the unbound CUR. The supernatant and washing solutions were collected, and the amount of free CUR was determined using the calibration curve. The amount of CUR loaded into the PDA particles was calculated by subtracting the amount of free CUR from the initially added CUR. The encapsulation efficiency and drug-loading capacity of PDA@CUR were calculated as follows [30]:

$$\mathrm{Encapsulation}\;\mathrm{efficiency}\;(\%)\;=\frac{m_{0}-m_{f}}{m_{0}}\;\times \;100\%$$

$$\mathrm{Drug}-\mathrm{loading}\;\mathrm{capacity}\;(\%)=\frac{m_{0}-m_{f}}{m_{PDA@CUR}}\times 100\%$$

where $m_{0}$ represents the initial mass of CUR added during preparation, $m_{f}$ represents the mass of free CUR remaining in the supernatant and washing solutions, and $m_{PDA@CUR}$ represents the dry mass of the obtained PDA@CUR particles.

### 2.5. Drug Loading

To determine the CUR content in PHMN/PDA@CUR, a known mass of dried PHMN/PDA@CUR was immersed in absolute ethanol and sufficiently disrupted to extract CUR. The samples were sonicated in a water bath sonicator for 30 min and shaken for 2 h to facilitate complete CUR extraction, followed by centrifugation to remove the insoluble microneedle matrix. The CUR concentration in the supernatant was determined at 425 nm. The CUR retention efficiency and drug-loading content of the microneedles were calculated as follows:

$$\mathrm{CUR}\;\mathrm{retention}\;\mathrm{efficiency}\;(\%)\;=\frac{m_{CUR,MN}}{m_{CUR,added}}\;\times \;100\%$$

$$\mathrm{CUR}\;\mathrm{content}\;\mathrm{in}\;\mathrm{microneedles}\;(\%)=\frac{m_{CUR,MN}}{m_{MN}}\times 100\%\;$$

where $m_{CUR,MN}$ is the mass of CUR extracted from the microneedles, $m_{CUR,added}$ is the amount of CUR introduced into the microneedle precursor solution, and $m_{MN}$ is the dry mass of the microneedles. For each determination, three independent tests were carried out.

### 2.6. In Vitro Drug Release

The in vitro release behavior of CUR from PHMN/PDA@CUR was evaluated under different pH conditions with or without NIR irradiation. Briefly, 10 mg of dried PHMN/PDA@CUR was directly immersed in 10 mL of phosphate-buffered saline (PBS) containing 1% (*v*/*v*) Tween 80 at pH 7.4 or 5.5. Tween 80 was included in the release medium to improve the aqueous solubility of CUR and facilitate maintenance of sink conditions during the release experiment. The samples were incubated at 37 °C under continuous shaking at 100 rpm in the dark. Four experimental conditions were established: pH 7.4, pH 7.4 + NIR, pH 5.5, and pH 5.5 + NIR. For the NIR-treated groups, the samples were irradiated once at the beginning of the release experiment using an 808 nm NIR laser at a power of 1.2 W for 10 min, and no additional NIR irradiation was applied during the subsequent release period. At predetermined time points (2, 4, 8, 12, 24, 48, and 72 h), 1 mL of release medium was withdrawn and immediately replaced with an equal volume of fresh release medium of the corresponding pH to maintain a constant total volume. The collected samples were centrifuged to remove suspended particles, and the CUR concentration in the supernatant was quantified by UV–visible spectrophotometry at 425 nm using the corresponding calibration curve. The cumulative amount of released CUR was corrected for medium replacement according to the following equation: Mt = Ct × V0 + Vs × ΣCi, where Mt is the cumulative amount of CUR released at time t, Ct is the CUR concentration in the release medium at time t, V0 is the total volume of the release medium, Vs is the volume withdrawn at each sampling point, and ΣCi is the sum of CUR concentrations in all previously withdrawn samples. The cumulative CUR release percentage was calculated as: Cumulative CUR release (%) = Mt/MCUR, loaded × 100%. All experiments were performed in triplicate (*n* = 3).

The pH 5.5 condition was selected as a mildly acidic experimental stimulus to evaluate the pH-responsive release characteristics of the PHMN/PDA@CUR system rather than as a universal representation of the infected-wound environment, because wound pH varies considerably with wound type and healing stage.

For the NIR-treated groups, the samples were irradiated once at the beginning of the release experiment using an 808 nm NIR laser at a power of 1.2 W for 10 min. No additional NIR irradiation was applied during the subsequent release period.

### 2.7. In Vitro Antibacterial Application

To evaluate the antibacterial efficiency of materials, a colony-forming unit (CFU) counting assay was carried out. Logarithmic-phase *E. coli* and *S. aureus* was diluted to approximately 1 × 10^6^ CFU mL^−1^ and co-incubated with samples from different groups in 96-well plates at 37 °C for 12 h. For the PHMN/PDA@CUR + NIR group, the samples were irradiated once immediately after mixing with the bacterial suspension using an 808 nm NIR laser at a power of 1.2 W for 10 min. No additional NIR irradiation was applied during the subsequent incubation. After that, 10-fold serial dilutions of the bacterial suspension were prepared using LB medium. Following serial dilution, 100 μL of each bacterial suspension was plated on LB agar and cultured at 37 °C for 16 h. After incubation, the number of viable colonies was determined, and the bacterial survival rate was calculated accordingly.

### 2.8. Cytocompatibility and Protection Against Oxidative Injury

The biocompatibility of PHMN/PDA@CUR was investigated by MTT and live/dead assays using two independent experimental sets. For the MTT assay, L929 fibroblasts were dispensed into 96-well plates at 1 × 10^4^ cells per well and allowed to adhere for 24 h. The original medium was subsequently replaced with fresh culture medium supplemented with PHMN/PDA@CUR extracts corresponding to PDA@CUR concentrations of 100, 200, 400, 800, and 1600 μg/mL. Untreated cells were used as the NC group. Following another 24 h incubation, 20 μL of MTT solution was introduced into each well to obtain a final concentration of 0.5 mg/mL, and the cells were further incubated for 3 h. The culture medium was then discarded, and the formed formazan products were solubilized in 100 μL of DMSO. Finally, the optical density of each well was recorded at 570 nm using a microplate reader. The mean absorbance of the NC group was normalized to 1, and relative cell viability was measured by this equation:

$$\mathrm{Relative}\;\mathrm{cell}\;\mathrm{viability}=\frac{A_{\mathrm{experimental}\;\mathrm{group}}}{A_{\mathrm{NC}\;\mathrm{group}}}$$

For live/dead staining, L929 fibroblasts (5 × 10^4^ cells/well) were separately seeded into 24-well plates. After culturing for 24 h, the cells were then independently treated with the corresponding PHMN/PDA@CUR extracts for another 24 h. After washing with PBS, the cells were stained with calcein-AM and propidium iodide according to the manufacturer’s instructions and incubated in the dark for 20 min. Fluorescence images were acquired using an inverted fluorescence microscope with a 10× objective lens. To evaluate protection against oxidative injury, L929 fibroblasts were separately cultured in 96-well plates and divided into the NC, H_2_O_2_, H_2_O_2_ + PHMN, H_2_O_2_ + PHMN/PDA@CUR, and H_2_O_2_ + PHMN/PDA@CUR+ NIR groups. After cell attachment, H_2_O_2_ at a final concentration of 0.1 mM and the corresponding microneedle extracts were simultaneously added to the designated groups. The equivalent PDA@CUR concentration was fixed at 400 μg/mL.

For the NIR-treated group, the PHMN/PDA@CUR extract was irradiated with an 808 nm laser (1.2 W) for 10 min and subsequently cooled to 37 °C before being added to the cells together with H_2_O_2_. After 24 h of co-incubation, the cell viability was measured with MTT assay as described above. The NC group was normalized to 1, and the data of other groups were analyzed based on the value of the NC group. Three independent tests were carried out for all samples.

### 2.9. Characterization Techniques

The physicochemical and structural properties of the prepared materials were characterized using standard analytical instruments. The chemical structures of HA and HAMA were analyzed with ^1^H-NMR (400 MHz, Bruker, Billerica, MA, USA) and Fourier transform infrared (FTIR, Nicolet iS10 spectrometer, Thermo Fisher Scientific, Madison, WI, USA) spectroscopy. The morphology of PDA and PDA@CUR nanoparticles was observed by transmission electron microscopy (TEM, Tecnai G2 F20, FEI Co., Hillsboro, OR, USA), while their elemental composition and crystalline structure were characterized by X-ray photoelectron spectroscopy (XPS, PHI-5000 VersaProbe-III spectrometer, ULVAC-PHI, Kanagawa, Japan) and X-ray diffraction (XRD, D8, Bruker, Billerica, MA, USA), respectively. The morphology and particle dimensions of PDA and PDA@CUR were determined from TEM images. Zeta potential was measured using a Nano ZSE analyzer (Malvern Instruments, Malvern, UK). UV–visible spectroscopy (UV-2600 spectrophotometer, Shimadzu, Suzhou, China) was used for CUR quantification and drug-release measurements.

The morphology and microstructure of PHMNs were measured with scanning electron microscopy (SEM, Hitachi Regulus 8100, Tokyo, Japan); before SEM observation, the dried samples were sputter-coated with a thin layer of gold to improve electrical conductivity and reduce charging effects, and their compressive mechanical properties (UTM5305H, Shenzhen Suns Technology Co., Ltd., Shenzhen, China) were evaluated using a universal mechanical testing machine. Photothermal experiments were performed using an 808 nm NIR laser, and temperature changes were recorded using an infrared thermal imaging camera (Fotric 223 s, Shanghai, China). Absorbance in the MTT assay was measured using a microplate reader (YP-96A, Shandong Youyunpu Optoelectronic Technology Co., Ltd., Jinan, China), while fluorescence images from the live/dead staining assay were acquired using a fluorescence microscope (CKX53SFC, Olympus, Shanghai, China).

### 2.10. Statistical Methods

Unless otherwise indicated, all quantitative data are presented as mean ± standard deviation (SD) from three independent experiments (*n* = 3). Statistical analyses were performed using GraphPad Prism (version 9.5.1). For endpoint comparisons involving more than two independent groups, one-way analysis of variance (ANOVA) followed by Tukey’s multiple-comparisons test was used. For time-course experiments involving both treatment condition and time, including CUR release and photothermal heating experiments, two-way repeated-measures ANOVA was performed, with treatment condition and time as the two factors, followed by Šídák’s multiple-comparisons test where appropriate. A value of *p* < 0.05 was considered statistically significant. In the figures, * *p* < 0.05, ** *p* < 0.01, *** *p* < 0.001, and **** *p* < 0.0001 indicate statistical significance, whereas “ns” denotes no significant difference.

## 3. Results and Discussion

### 3.1. Synthesis and Physicochemical Characterizations of PDA@CUR Nanoparticles

**Figure 2:**
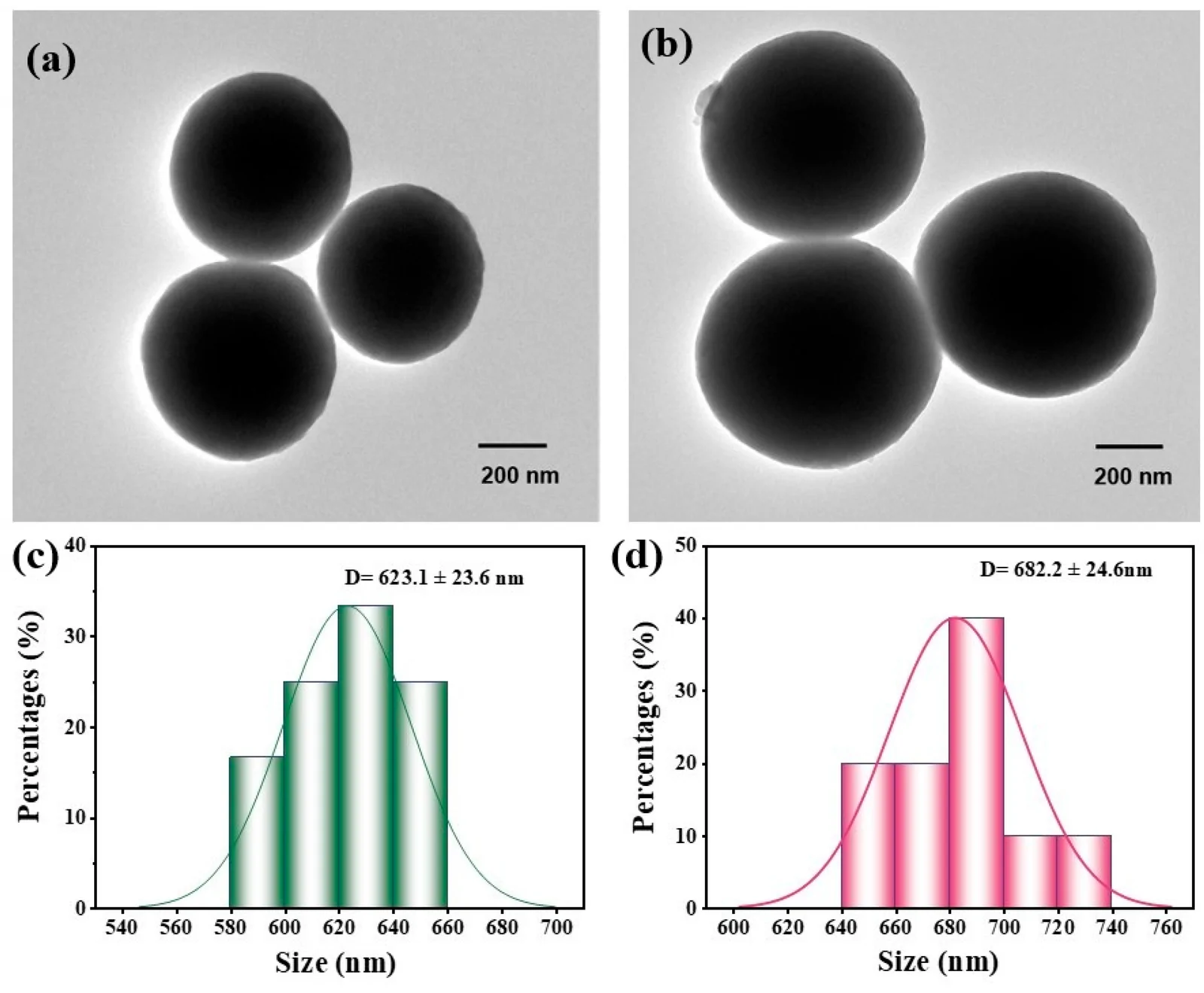
Morphological characterization and particle-size distributions of PDA and PDA@CUR nanoparticles. (**a**,**b**) TEM images of (**a**) PDA and (**b**) PDA@CUR particles; scale bars: 200 nm. (**c**,**d**) Particle-size distributions of (**c**) PDA and (**d**) PDA@CUR nanoparticles. The bars represent the measured particle-size frequency distribution, and the curves indicate the corresponding Gaussian fitting curves.

PDA nanoparticles can be readily synthesized via the self-oxidative polymerization and self-assembly of polymer monomers under mildly alkaline system [25]. In an aqueous environment, dopamine undergoes oxidation to quinone intermediates, followed by intramolecular cyclization, polymerization, and π–π stacking interactions. These processes promote the nucleation and gradual growth of PDA into stable nanoparticles. The morphology and particle size of PDA before and after CUR loading were first examined by TEM. As presented in Figure 2a, the synthesized PDA particles exhibited a relatively uniform spherical morphology with smooth and well-defined boundaries. After CUR incorporation, the PDA@CUR particles retained an overall spherical morphology without obvious structural collapse or severe aggregation (Figure 2b), suggesting that CUR loading did not substantially alter the overall morphology of the PDA particles.

TEM-based image analysis further revealed an increase in particle dimensions after CUR incorporation. The mean particle diameter of pristine PDA was 623.1 ± 23.6 nm (Figure 2c), whereas that of PDA@CUR increased to 682.2 ± 24.6 nm (Figure 2d), corresponding to an approximately 9.5% increase in mean particle size. The increase in particle size while maintaining the overall spherical morphology is consistent with the incorporation of CUR into/onto the PDA particles. Together with the subsequent physicochemical characterization, these results support the successful preparation of CUR-loaded PDA nanoparticles.

### 3.2. Drug-Loading Performance and Ph-Responsive Release

**Figure 3:**
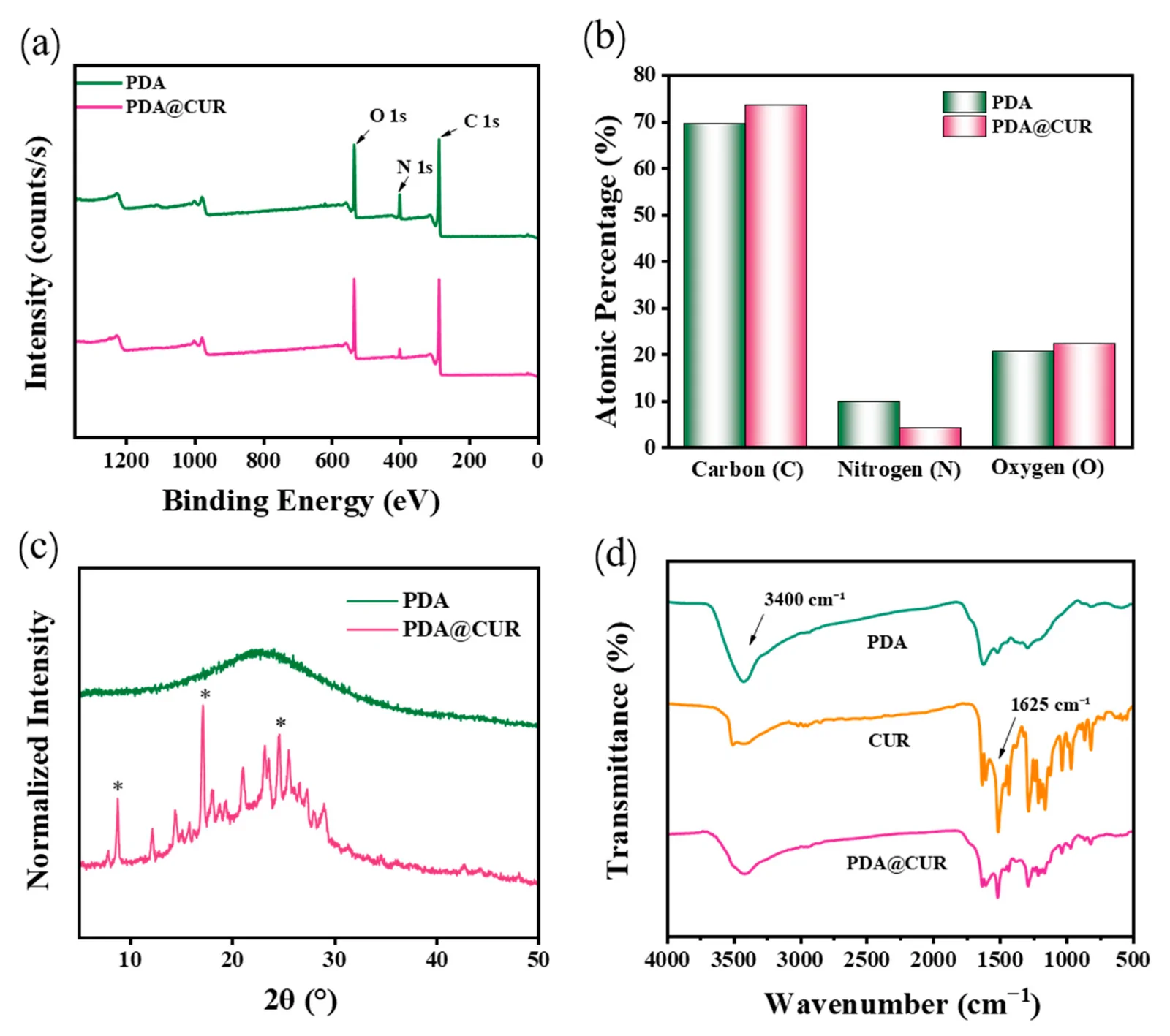
Chemical and structural characterizations of PDA and PDA@CUR. (**a**) XPS survey spectra of PDA and PDA@CUR. (**b**) Relative atomic percentages of carbon, nitrogen, and oxygen determined from the XPS spectra. (**c**) XRD patterns of PDA and PDA@CUR. (**d**) FTIR spectra of PDA, CUR, and PDA@CUR. * indicates the characteristic peaks of CUR.

To further verify the successful incorporation of CUR into PDA particles, the chemical composition and structural characteristics of PDA and PDA@CUR were analyzed. It can be found that the XPS survey spectra of both PDA and PDA@CUR exhibit characteristic C 1s, N 1s, and O 1s signals (Figure 3a,b). After CUR loading, the relative C content increased, whereas the N content decreased markedly, accompanied by a slight change in the O content. Since CUR is rich in C- and O-containing groups but contains no N, this alteration in surface elemental composition agrees well with the presence of CUR within or on the surface of PDA particles.

XRD analysis further reveals distinct structural differences between PDA and PDA@CUR (Figure 3c). PDA displayed a broad diffraction peak centered at approximately 20–25°, indicative of its predominantly amorphous structure [31]. In contrast, PDA@CUR exhibited several sharper diffraction peaks superimposed on the broad PDA background. These peaks were attributable to the crystalline characteristics of CUR, suggesting that CUR was retained within the composite while maintaining part of its crystalline structure [32]. Meanwhile, FTIR spectra provide additional evidence for the formation of PDA@CUR (Figure 3d). PDA exhibited a broad absorption band around 3400 cm^−1^, mainly associated with O–H and N–H stretching vibrations [33]. CUR displayed characteristic absorption features, including a prominent band around 1625 cm^−1^. After CUR loading, characteristic spectral features originating from both PDA and CUR were retained in the PDA@CUR spectrum, with slight changes in the band shape and intensity. Together with the TEM and XPS results, these XRD and FTIR findings support the successful formation of the PDA@CUR composite, most likely through noncovalent interactions such as π–π stacking, hydrogen bonding, and hydrophobic interactions rather than the formation of new covalent bonds.

**Figure 4:**
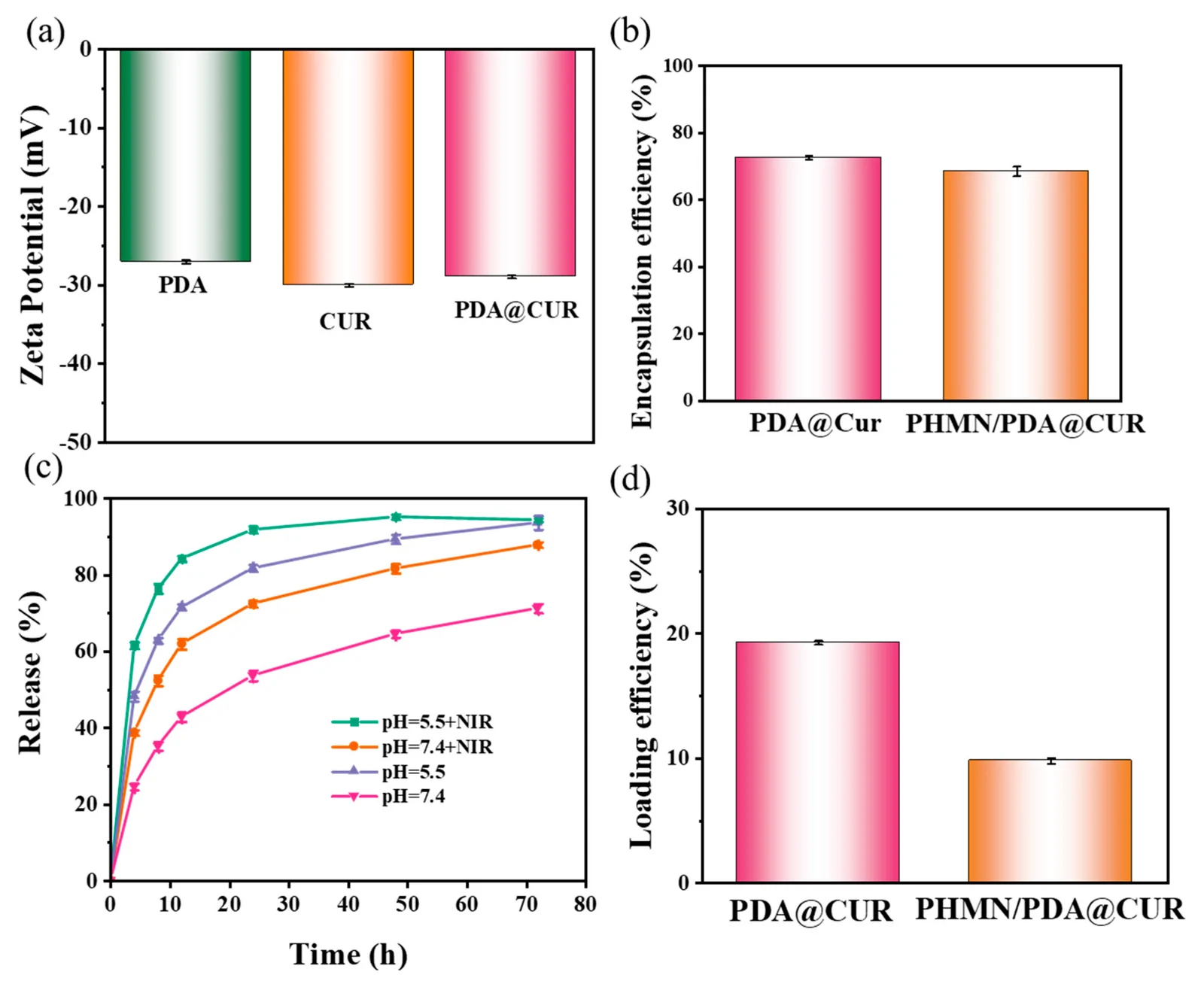
Drug-loading performance and responsive CUR release of the PHMN/PDA@CUR microneedle system. (**a**) Zeta potentials of PDA, CUR, and PDA@CUR. (**b**) Encapsulation efficiency of CUR in PDA@CUR and CUR retention efficiency after microneedle fabrication. (**c**) Cumulative CUR release profiles under pH 5.5 and 7.4 conditions with or without NIR irradiation. (**d**) Drug-loading content of PDA@CUR and PHMN/PDA@CUR. Data are presented as mean ± SD (*n* = 3).

Zeta potential analysis demonstrates that the surface potentials of PDA, CUR, and PDA@CUR were all negative and remained around −30 mV (Figure 4a). The absence of a pronounced change in zeta potential after CUR loading indicates that CUR incorporation did not substantially alter the negatively charged surface characteristics of PDA. Meanwhile, the relatively high absolute zeta-potential values suggest favorable electrostatic stability of the resulting dispersions.

The drug-loading performance of the system was subsequently evaluated. It is clear that the encapsulation efficiency of CUR in PDA@CUR was approximately 72%, and 68% of the initially incorporated CUR was retained after fabrication into the microneedle system (Figure 4b). The drug-loading content of PDA@CUR reached approximately 19%, whereas the CUR loading content of the final microneedles was approximately 10% (Figure 4d). The reduction observed after microneedle fabrication can be attributed to the introduction of the HAMA/PVA polymer matrix, which increased the total mass of the formulation while preserving a substantial proportion of the loaded drug. These results demonstrate that PDA provides an effective carrier for CUR and that most of the drug can be retained during subsequent incorporation into the PHMN platform.

The release behavior of CUR from the PHMN/PDA@CUR system exhibited clear dependence on both environmental pH and NIR irradiation (Figure 4c). Under physiological conditions (pH 7.4), approximately 70% of CUR was cumulatively released within 72 h. NIR irradiation accelerated drug release under the same pH condition, resulting in a cumulative release approaching 90%. A more rapid release profile was observed under mildly acidic conditions (pH 5.5), with the cumulative release exceeding 90% over the experimental period. The combination of acidic conditions and NIR irradiation produced the fastest initial release, reaching approximately 80% within the early stage and approaching 95% at 72 h.

The enhanced CUR release under acidic conditions may be associated with weakening of the noncovalent interactions between CUR and PDA and altered interactions between the particles and the surrounding polymer network [18]. In addition, photothermal heating generated by PDA under NIR irradiation may enhance molecular mobility and diffusion, thereby accelerating CUR desorption and release. Notably, although NIR irradiation markedly accelerated the early release rate, its effect on the final cumulative release was less pronounced under pH 5.5, where CUR release was already extensive. Taken together, these results indicate that the PHMN/PDA@CUR presents a pH- and NIR-responsive release profile, enabling accelerated drug liberation under acidic and photothermal stimulation while maintaining comparatively slower release under physiological conditions.

### 3.3. Morphological and Mechanical Properties of PHMN/PDA@CUR

**Figure 5:**
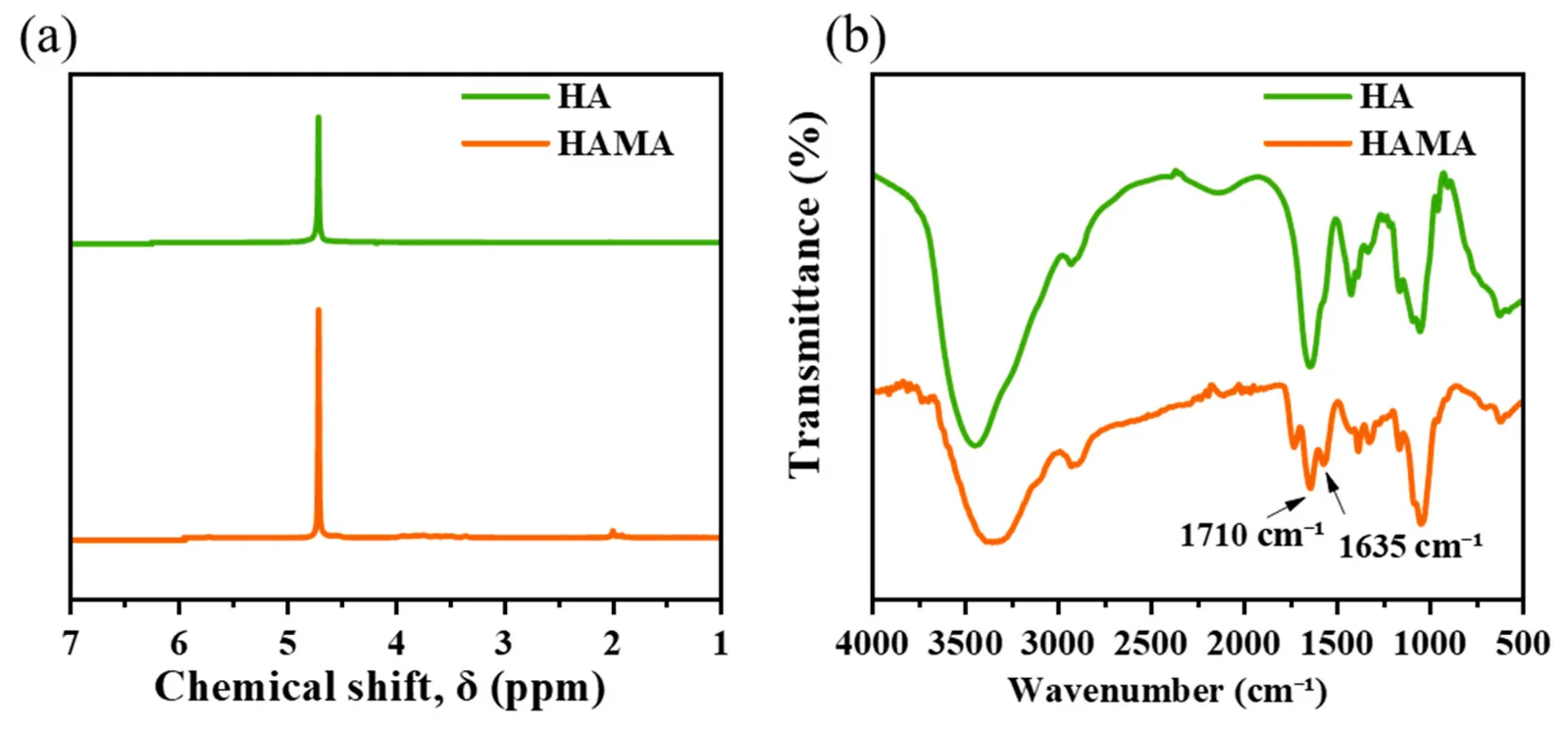
Chemical characterization of HA and HAMA. (**a**) ^1^H NMR spectra; (**b**) FTIR spectra.

The successful methacrylation of HA was first verified by ^1^H NMR and FTIR spectroscopy. Compared with native HA, the ^1^H NMR spectrum (Figure 5a) of HAMA exhibited additional characteristic signals attributable to the introduced methacrylate groups, particularly the vinyl proton signals at approximately 5.6–6.1 ppm, confirming the successful introduction of methacryloyl functionalities into the HA backbone [34].

As shown in Figure 5b, FTIR analysis further supports the chemical modification of HA. Both HA and HAMA exhibited a broad absorption band at approximately 3200–3500 cm^−1^, which is ascribed to the stretching vibration of hydroxyl groups in the polysaccharide backbone. Compared with native HA, HAMA exhibited a distinct absorption band at approximately 1710 cm^−1^, corresponding to the C=O stretching vibration of the introduced methacrylate ester groups. In addition, the absorption band around 1635 cm^−1^ was associated with the unsaturated C=C functionality and overlapped partially with the characteristic amide/carboxylate-related vibrations of HA [35]. The appearance and enhancement of these methacrylate-related absorption features, together with the characteristic vinyl proton signals observed by ^1^H NMR, confirmed the successful methacrylation of HA. Taken together, the ^1^H NMR and FTIR results verified the successful synthesis of HAMA, providing a photocrosslinkable polymer matrix for subsequent microneedle fabrication.

The chemical modification and degree of methacrylation (DM) of HAMA were evaluated by 1H NMR spectroscopy using D2O as the deuterated solvent. The characteristic vinyl proton signals of the introduced methacrylate groups were observed at approximately 5.6–6.2 ppm, whereas the N-acetyl methyl proton signal of the HA backbone at approximately 1.9–2.0 ppm was used as the internal reference. The DM was calculated from the relative integrated peak areas according to the following equation: DM (%) = [(Ivinyl/2)/(IN-acetyl/3)] × 100%, where Ivinyl represents the total integrated area of the methacrylate vinyl proton signals and IN-acetyl represents the integrated area of the N-acetyl methyl proton signal of HA. Based on the 1H NMR integration analysis, the DM of the synthesized HAMA was approximately 50%.

**Figure 6:**
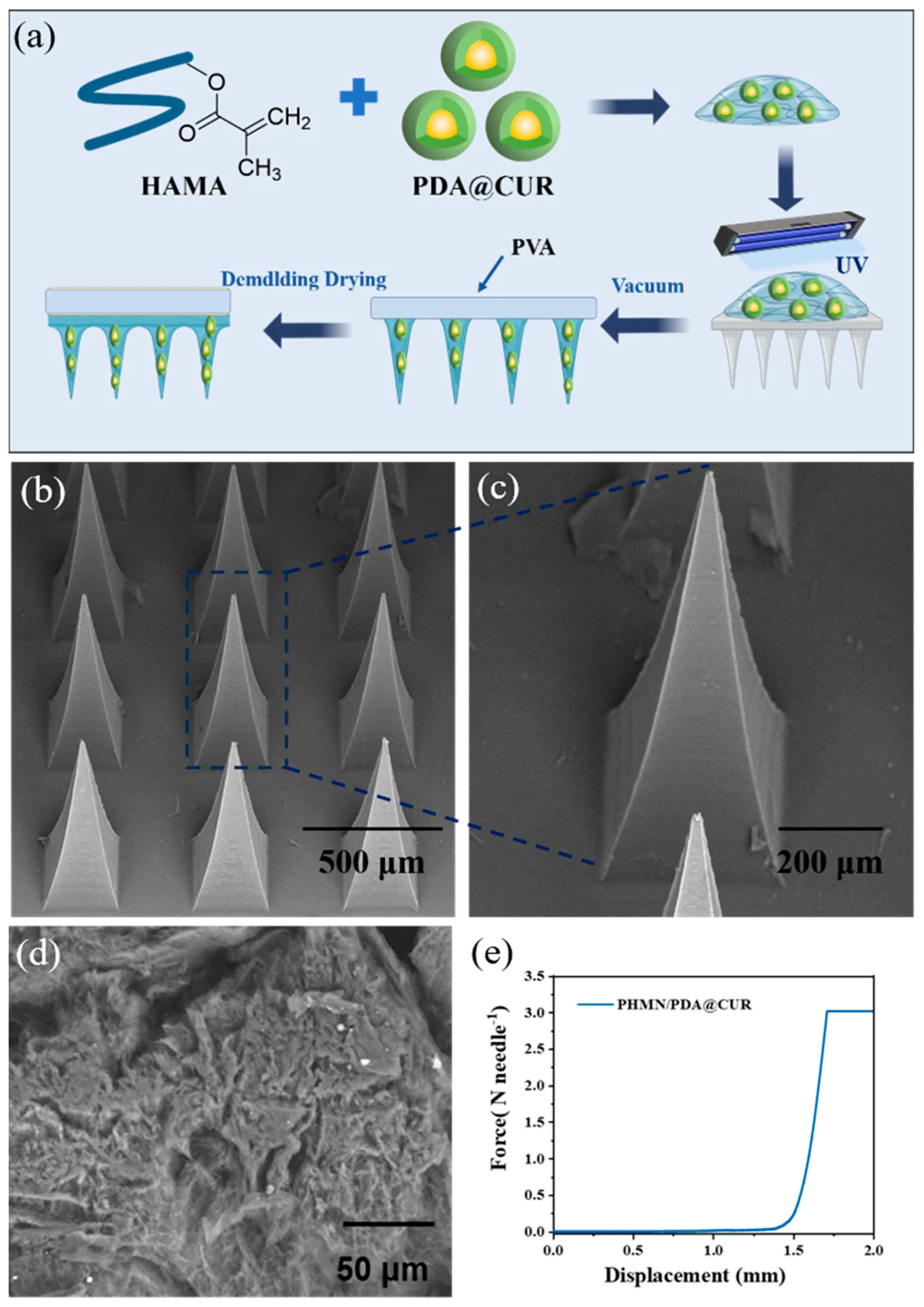
Fabrication, morphology, and mechanical properties of PDA@CUR-loaded polymeric microneedles. (**a**) Schematic presentation of the fabrication of PHMN/PDA@CUR. Image was created by Microsoft Office PowerPoint 2019. (**b**) SEM image of the microneedle array. (**c**) Magnified SEM image showing the morphology of an individual microneedle. (**d**) SEM image showing the microstructure of the microneedle matrix. (**e**) Representative force–displacement curve of PHMN/PDA@CUR under compression.

The PHMN/PDA@CUR was fabricated using a two-layer molding strategy, as schematically illustrated in Figure 6a. PDA@CUR particles were incorporated into the HAMA precursor solution and introduced into the microneedle cavities under vacuum, followed by UV-induced crosslinking to form the functional needle tips. A PVA layer was subsequently introduced as the backing layer, and the microneedle patches were obtained after drying and demolding [36]. This configuration enabled PDA@CUR to be incorporated predominantly within the HAMA needle region while PVA provided structural support for handling and application.

SEM observations demonstrated that the fabricated PHMN/PDA@CUR arrays possessed a well-organized and relatively uniform morphology (Figure 6b). Individual microneedles exhibited a pyramidal geometry with sharp and intact tips, and no obvious deformation or large-scale structural collapse was observed throughout the array. Higher-magnification imaging further confirmed the well-defined tapered structure of individual microneedles (Figure 6c), which is favorable for concentrating the applied force at the needle tip during insertion.

The microstructure of the microneedle matrix was then observed with SEM analysis (Figure 6d). A relatively rough and heterogeneous internal morphology was observed, suggesting the formation of a crosslinked polymeric network containing dispersed PDA@CUR particles. Such a structure may facilitate hydration and diffusion of the incorporated therapeutic cargo after contact with an aqueous environment while maintaining the overall structural integrity of the microneedles.

Adequate mechanical strength is essential for microneedles to resist deformation during application. As shown in Figure 6e, the compressive force increased progressively with displacement and rose sharply at larger deformation. The maximum recorded compressive force approached approximately 3.0 N/needle, indicating that the fabricated PHMN/PDA@CUR microneedles could withstand substantial compressive loading without immediate structural failure. Together with their sharp and intact morphology, these findings demonstrate the mechanical robustness of the prepared microneedles. However, direct skin-insertion or penetration experiments were not performed in the present study. Therefore, the compression results should be interpreted as evidence of mechanical robustness rather than direct confirmation of transdermal penetration capability. Further studies using ex vivo skin or validated skin-mimicking models will be required to directly evaluate insertion efficiency and penetration depth.

### 3.4. Photothermal Properties of PHMN/PDA@CUR

**Figure 7:**
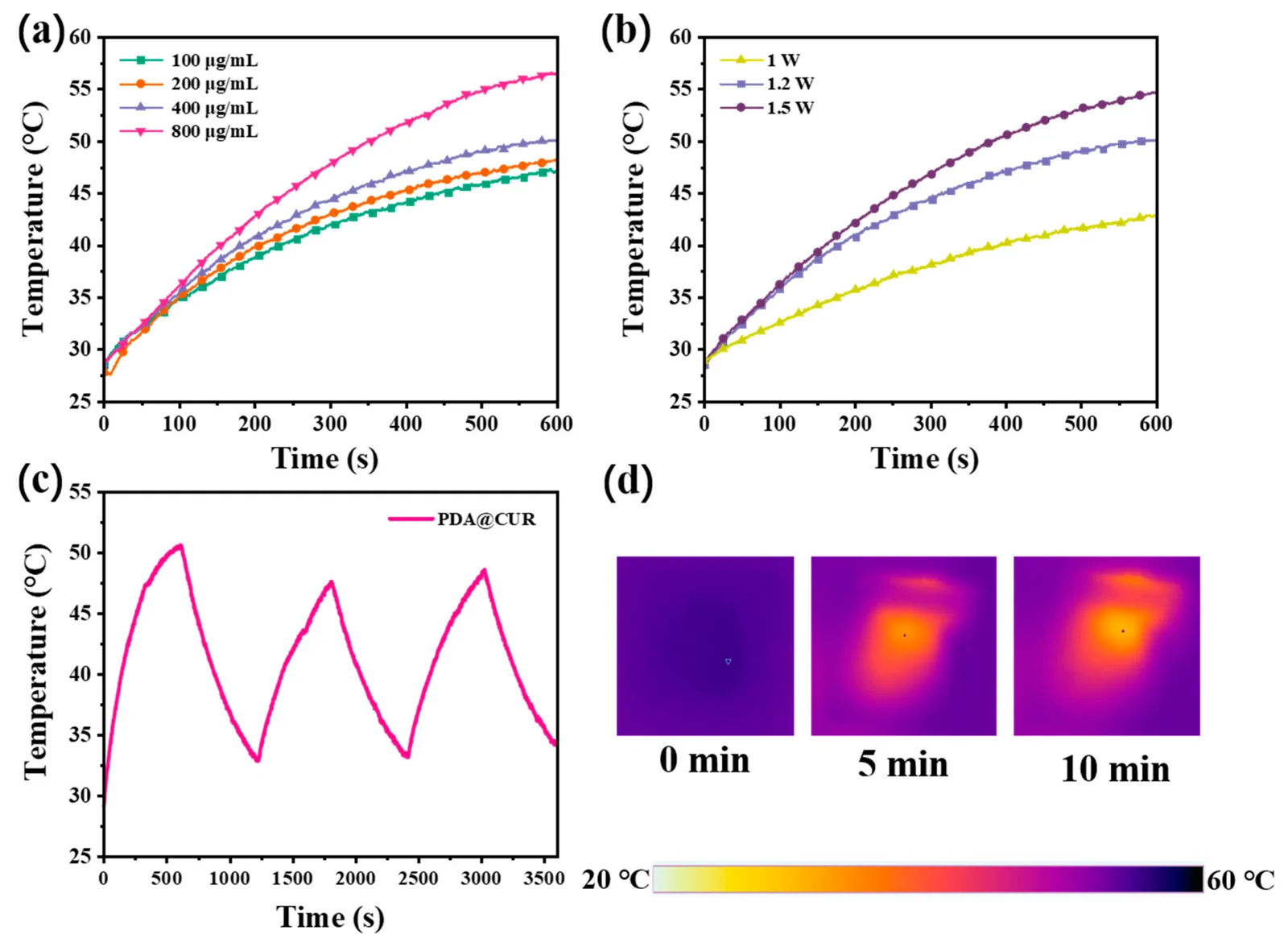
Photothermal properties of PDA@CUR and PHMN/PDA@CUR under NIR irradiation. (**a**) T–t profiles of PDA@CUR at various concentrations. (**b**) Temperature–time profiles of PDA@CUR under various NIR irradiation powers. (**c**) Photothermal heating–cooling curves of PDA@CUR over three consecutive irradiation cycles. (**d**) Representative infrared thermal images of the PHMN/PDA@CUR microneedle patch after NIR irradiation for 0, 5, and 10 min, respectively.

The photothermal performance of the PDA@CUR-containing system was systematically evaluated under NIR irradiation. As shown in Figure 7a, PDA@CUR exhibited a clear concentration-dependent photothermal response. Upon continuous NIR irradiation for 10 min, the temperature of all groups gradually increased, and a higher PDA@CUR concentration resulted in a greater temperature elevation [20]. The final temperatures of the 100, 200, 400, and 800 μg/mL groups reached approximately 47, 48, 50, and 56 °C, respectively. These results demonstrate that the photothermal heating behavior of PDA@CUR could be readily modulated by changing the material concentration, which is attributable to the broad NIR absorption and pronounced photothermal heating capability of PDA.

The influence of irradiation power was further examined at a fixed PDA@CUR concentration of 400 μg/mL. As shown in Figure 7b, the temperature elevation increased progressively with increasing laser power. After 10 min of irradiation, the sample temperatures reached approximately 43, 50, and 55 °C at laser powers of 1.0, 1.2, and 1.5 W, respectively. Thus, both material concentration and irradiation power could effectively regulate the photothermal response of the PDA@CUR system. Based on the concentration- and power-dependent heating profiles, 400 μg/mL PDA@CUR and 1.2 W NIR irradiation were selected as the working conditions for subsequent photothermal experiments.

The photothermal reproducibility of PDA@CUR was subsequently assessed through three consecutive irradiation–cooling cycles (Figure 7c). A rapid temperature increase was observed during each irradiation period, followed by a gradual decrease after withdrawal of the NIR stimulus. Although a slight reduction in the peak temperature occurred after the first cycle, the subsequent heating profiles remained generally reproducible, indicating that PDA@CUR retained a stable photothermal response during repeated NIR irradiation.

To further verify whether the photothermal capability of PDA@CUR was retained after incorporation into the PHMN matrix, the macroscopic thermal response of PHMN/PDA@CUR was observed using an infrared thermal imaging camera, as shown in Figure 7d. Before NIR irradiation, the microneedle patch exhibited a relatively low and uniform thermal signal. After 5 min of irradiation, an obvious increase in surface temperature was observed, and the high-temperature region became more pronounced after 10 min. These results demonstrate that incorporation of PDA@CUR into the HAMA/PVA microneedle matrix did not abolish its photothermal activity and that the resulting PHMN/PDA@CUR patch could efficiently generate localized heat under NIR irradiation.

Although the selected irradiation condition produced a pronounced photothermal response, the temperature approached approximately 50 °C after 10 min of NIR irradiation. Such temperatures may potentially cause thermal injury if directly generated at the tissue interface. In addition, direct cytocompatibility under simultaneous PHMN/PDA@CUR exposure and NIR irradiation was not evaluated in the present study. Therefore, further optimization of irradiation intensity, exposure duration, and local temperature control will be required before in vivo application to achieve an appropriate balance between antibacterial efficacy and thermal safety.

Collectively, the concentration- and power-dependent heating behavior, reproducible photothermal cycling, and evident thermal response of the final microneedle patch confirmed the effective NIR-responsive photothermal properties of the system, providing the basis for subsequent photothermally enhanced antibacterial experiments.

### 3.5. NIR-Enhanced Antibacterial Activity

**Figure 8:**
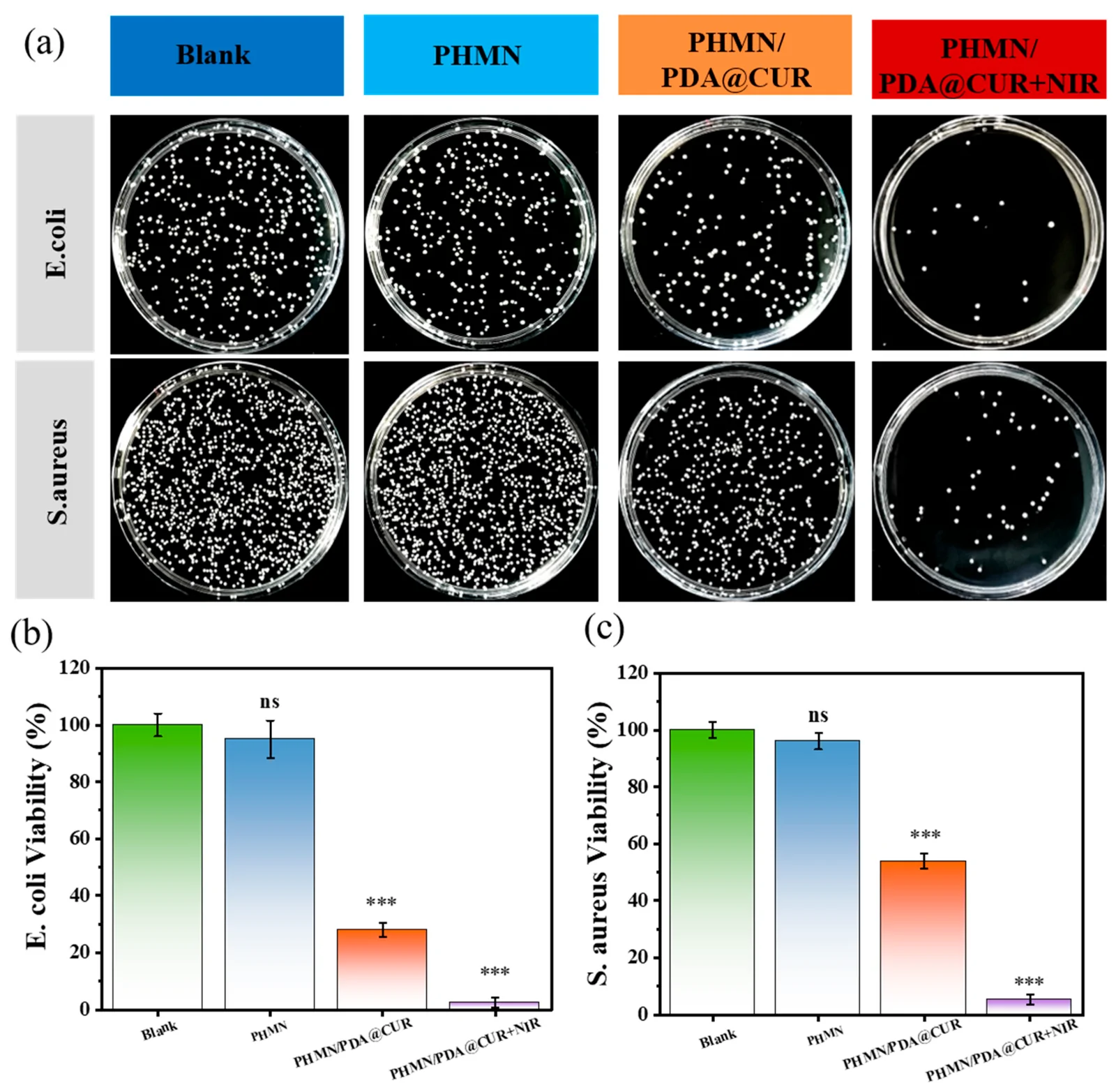
NIR-enhanced antibacterial efficacy of PHMN/PDA@CUR. (**a**) Representative agar-plate images of *E. coli* and *S. aureus* after corresponding treatments. (**b**) Relative viability of *E. coli* after treatment with Blank, PHMN, PHMN/PDA@CUR, and PHMN/PDA@CUR + NIR. (**c**) Relative viability of *S. aureus* after the corresponding treatments. Data are presented as mean ± SD (*n* = 3). Statistical significance was determined using one-way ANOVA followed by Tukey’s multiple comparisons test. *** *p* < 0.001, and ns indicates no significant difference. The comparisons indicated by the asterisks are between the indicated groups.

The antimicrobial efficacy of PHMN/PDA@CUR was examined using *E. coli* as a representative Gram-negative bacterium and *S. aureus* as a representative Gram-positive bacterium. Bacterial viability was quantified by a CFU assay. As illustrated in Figure 8a, extensive colony formation was detected in the untreated controls for both bacterial species [37]. Treatment with blank sample and PHMN resulted in only a slight reduction in colony numbers, whereas incorporation of PDA@CUR markedly decreased bacterial growth. Notably, NIR irradiation further enhanced the antibacterial effect of PHMN/PDA@CUR, with only a few residual colonies observed on the agar plates.

Quantitative analysis was consistent with the representative colony images. For *E. coli*, bacterial viability remained close to that of the Blank group after PHMN treatment, with no statistically significant difference (Figure 8b). In contrast, PHMN/PDA@CUR reduced bacterial viability to approximately 30%, demonstrating that incorporation of PDA@CUR endowed the microneedles with substantial intrinsic antibacterial activity. Upon NIR irradiation, bacterial viability was further reduced to only a few percent, indicating a pronounced enhancement of antibacterial efficacy.

A similar trend was observed for *S. aureus* (Figure 8c). PHMN showed no significant antibacterial activity compared with the Blank group, whereas PHMN/PDA@CUR reduced bacterial viability to approximately 50%. After NIR irradiation, bacterial viability decreased dramatically to below 10%, confirming the strong NIR-enhanced antibacterial effect of the PDA@CUR-containing microneedles.

The antibacterial activity observed in the absence of NIR may primarily arise from CUR, which has been reported to interfere with bacterial membrane integrity and cellular functions. Following NIR irradiation, PDA efficiently converted light energy into heat, producing a localized photothermal effect capable of inducing bacterial membrane and protein damage. Meanwhile, the increased temperature may also accelerate CUR release from the microneedle system, thereby increasing local drug availability. Consequently, the combination of CUR-mediated antibacterial activity and PDA-mediated photothermal treatment produced a substantially greater antibacterial effect than PDA@CUR treatment alone.

The PHMN/PDA@CUR patch exhibited antibacterial activity against both *E. coli* and *S. aureus*, and NIR irradiation further enhanced this antibacterial effect. The antibacterial activity observed in the absence of NIR may be associated with the reported antibacterial properties of CUR, whereas the enhanced activity after NIR irradiation may involve PDA-mediated photothermal heating together with NIR-promoted CUR release. However, because component-specific control groups were not included in the present study, the individual contributions of CUR and PDA-mediated photothermal effects cannot be quantitatively distinguished. Therefore, the present results support an NIR-enhanced antibacterial effect of the integrated PHMN/PDA@CUR system rather than a definitive mechanistic attribution to individual components.

It should be noted that the present antibacterial evaluation was performed using planktonic *E. coli* and *S. aureus* rather than established bacterial biofilms. Therefore, the current results should not be interpreted as direct evidence of antibiofilm activity, and further studies using established biofilm models are required to evaluate the antibiofilm potential of the PHMN/PDA@CUR system.

### 3.6. Cytocompatibility and Protection from H_2_O_2_-Induced Injury

**Figure 9:**
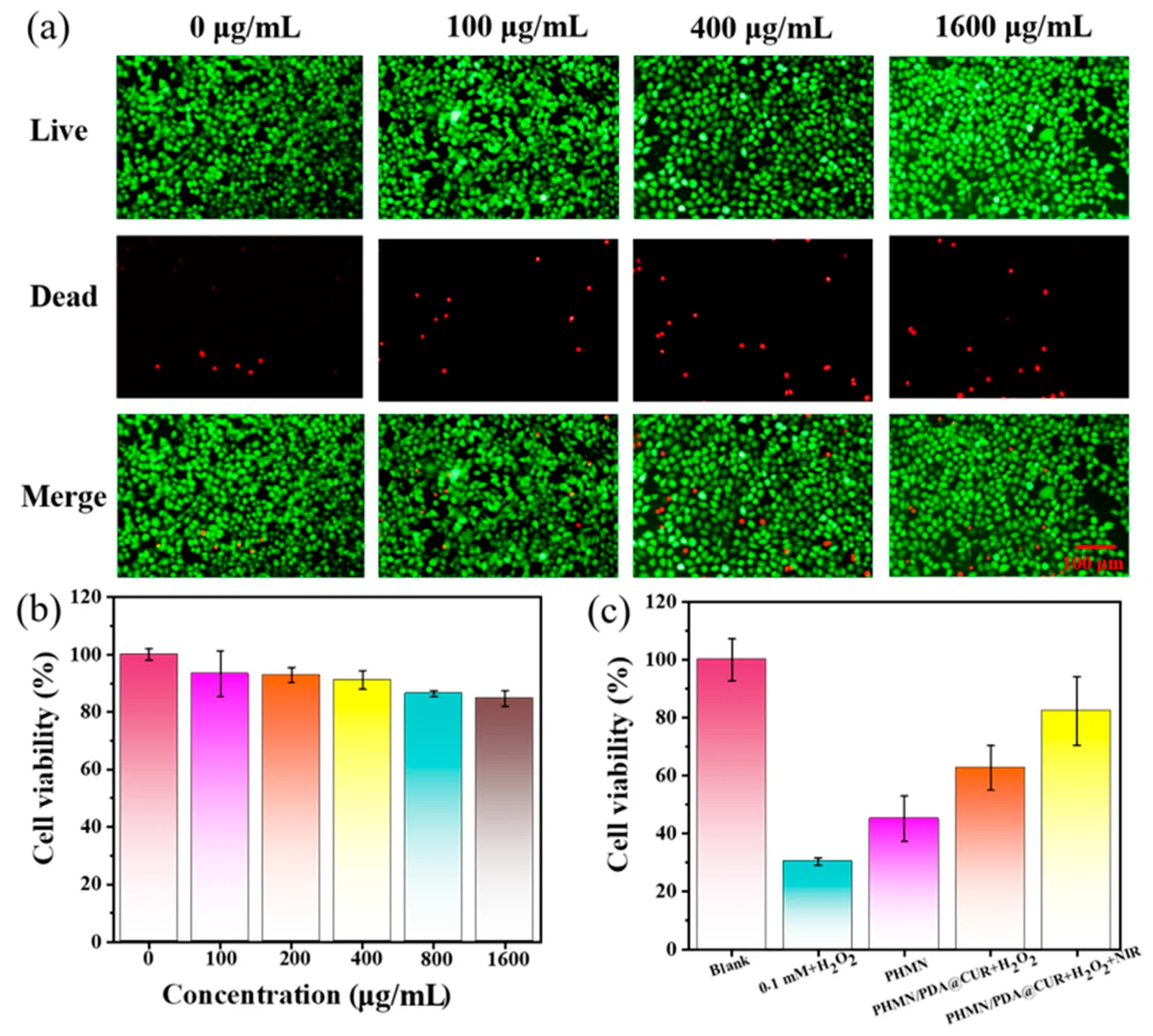
Cytocompatibility and protective effect of PHMN/PDA@CUR against H_2_O_2_-induced oxidative injury. (**a**) Representative live/dead fluorescence images of L929 fibroblasts treated with different concentrations of PDA@CUR-containing microneedle extracts. Live cells were visualized by green fluorescence, whereas dead cells were identified by red fluorescence. (Scale bars: 100 μm.) (**b**) Relative cell viability of L929 fibroblasts after treatment with different equivalent concentrations of PDA@CUR, as determined by the MTT assay. (**c**) Relative cell viability of L929 fibroblasts subjected to H_2_O_2_-induced oxidative injury after different treatments. Data are presented as mean ± SD (*n* = 3).

The cytocompatibility of PHMN/PDA@CUR was first evaluated using L929 fibroblasts. Live/dead staining showed that the majority of cells remained viable after treatment with different concentrations of the material (Figure 9a). Strong green fluorescence was observed in all groups, whereas only a small number of dead cells with red fluorescence were detected. No obvious concentration-dependent increase in cell death was observed even at a PDA@CUR concentration of 1600 μg/mL, indicating favorable cytocompatibility over the tested concentration range.

The MTT results were consistent with the live/dead staining observations (Figure 9b). Cell viability remained above approximately 80% throughout the concentration range of 100–1600 μg/mL. The metabolic activity remained relatively stable across the tested concentration range, despite a modest concentration-dependent decline. These results indicate that the PHMN/PDA@CUR system exhibited acceptable cytocompatibility and provide support for the use of 400 μg/mL as the working concentration in subsequent experiments.

The protective effect of the PHMN/PDA@CUR system against oxidative injury was subsequently investigated using an H_2_O_2_-induced cell damage model. As shown in Figure 9c, exposure to 0.1 mM H_2_O_2_ markedly reduced cell viability to approximately 30% of the untreated control, confirming successful induction of oxidative injury [38]. Treatment with PHMN increased cell viability to approximately 45%, whereas PHMN/PDA@CUR further restored cell viability to approximately 60–65%, demonstrating a clear protective effect of the PHMN/PDA@CUR against H_2_O_2_-induced cellular damage.

Notably, the PHMN/PDA@CUR + NIR group exhibited the highest cell viability among the H_2_O_2_-treated groups, reaching approximately 80% of the control level. In this experiment, the PDA@CUR-containing treatment solution was irradiated with NIR before being cooled and applied to the cells; therefore, the improved cellular protection cannot be attributed to direct photothermal heating of the cells. Instead, the enhanced effect is more reasonably associated with NIR-promoted release of CUR from the PDA@CUR system, thereby increasing the availability of CUR during H_2_O_2_ exposure. Given the well-documented antioxidant and cytoprotective properties of CUR, the increased drug availability may contribute to improved cellular tolerance to oxidative stress.

Therefore, we suggest that PHMN/PDA@CUR exhibited favorable cytocompatibility over a broad concentration range and effectively attenuated H_2_O_2_-induced loss of cell viability. NIR pretreatment further enhanced this protective effect, consistent with the NIR-responsive drug-release behavior demonstrated above. These findings indicate that the developed PHMN/PDA@CUR system not only possesses antibacterial photothermal functionality but also provides a favorable cellular compatibility profile and protection against oxidative injury.

## 4. Conclusions

In this study, a PDA@CUR-loaded HAMA/PVA microneedle system was developed for localized antibacterial and cytoprotective treatment. PDA served as both a CUR carrier and an NIR-responsive photothermal component, while the HAMA/PVA microneedle architecture provided an effective platform for localized delivery. The resulting PHMN/PDA@CUR microneedles exhibited pH- and NIR-responsive CUR release, efficient photothermal performance, enhanced antibacterial activity against *E. coli* and *S. aureus*, and favorable cytocompatibility. Moreover, the system partially protected fibroblasts against H_2_O_2_-induced oxidative injury. Overall, this multifunctional microneedle platform provides an in vitro proof of concept for integrating localized CUR delivery with PDA-mediated photothermal antibacterial treatment and warrants further investigation for infected wound management.

## Data Availability

The data shown in this study can also be obtained by requesting to the corresponding authors.
